# D‐Chiro Inositol Supplementation and the Occurrence of Gestational Diabetes: A Randomized Controlled Trial in China

**DOI:** 10.1002/fsn3.4601

**Published:** 2024-12-05

**Authors:** Xinning Chen, Zhimei Du, Zhanwei Zhang, Danqing Chen

**Affiliations:** ^1^ Department of Obstetric, Women's Hospital Zhejiang University School of Medicine Hangzhou Zhejiang China; ^2^ Department of Obstetric Quzhou Maternal and Children's Hospital Quzhou Zhejiang China; ^3^ School of Medicine Zhejiang University Hangzhou Zhejiang China

**Keywords:** D‐chiro inositol, gestational diabetes, prevention

## Abstract

To investigate the effect of D‐chiro inositol (DCI) supplementation on perinatal outcomes in pregnant women at high risk of gestational diabetes mellitus (GDM), we conducted a prospective, randomized, placebo‐controlled study. Eligibility criteria included women aged ≥ 35 years old, with a pre‐pregnancy body mass index ≥ 24 kg/m^2^, having a family history of type 2 diabetes, having a history of GDM, polycystic ovary syndrome, or a history of delivering macrosomia infants. Participants who were recruited at a gestational age of 12–16 weeks, were randomly to receive either DCI 500 mg twice daily or to receive a placebo for 12 weeks. Outcome measured included the occurrence of GDM and other perinatal outcomes. Between 2020 and 2022, 276 participants were enrolled, with 139 in the DCI Group and 137 in the Control Group. Occurrence of GDM was significantly lower in the DCI group compared to that in placebo group (24.8% vs. 38.1%, *p* = 0.027). A significant difference was observed in the 1‐h glycemia during the oral glucose tolerance test (OGTT) (8.35 ± 1.55 vs. 8.81 ± 1.85, *p* = 0.043), however, no significant differences in the fasting glucose level or 2‐h glycemia between the two groups. The mean birth weight of newborns in the control group was significantly heavier than in the DCI group (3487.9 ± 437.7 g vs. 3341.6 ± 420.1 g, *p* = 0.011). Therefore, DCI supplementation in early pregnancy can reduce the occurrence of GDM in women at high risk.

**Trial Registration:** ClinicalTrials.gov identifier: NCT **04801485**

## Graphical abstract

It's a prospective, randomized placebo‐controlled study in pregnant women with high risk for gestational diabetes. D‐chiro inositol supplementation in the second trimester is associated with decreased occurrence of GDM and a lower mean birth weight of newborns.

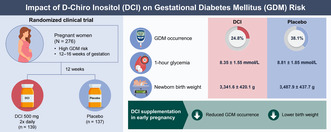

## 1 Introduction

Gestational diabetes mellitus (GDM) is associated with a higher risk of adverse perinatal outcomes, including preterm birth, macrosomia, stillbirths, etc., as well as a potential risk of future metabolic disorders in both mothers and offspring (Farrar et al. 2016; Lemieux et al. 2022; Lowe Jr. et al. 2019, 2018). It is estimated that about 1%–30% pregnancies are affected by gestational diabetes because of differences in race and non‐unified diagnostic and screening criteria (McIntyre et al. 2019). The incidence of GDM is rising, partially owing to increasing maternal age, as advanced maternal age is related with a high risk of GDM (Lee et al. 2023; Zhu et al. 2022).

Being overweight, smoking, having a family history of diabetes, and having a history of GDM are other identified risk factors for GDM according to previous studies (Zhang et al. 2021). Early recognition of high‐risk populations and targeted management are of great benefit for reducing the burden of GDM. Appropriate diet, physical activity, metformin, and special supplements are well‐established preventive approaches (Davenport et al. 2018; Doi et al. 2020; Yin et al. 2020). Inositol, a carbocyclic polyol, has been found to have insulin‐like properties and is thought to be efficient in improving glycemic control. A few studies have demonstrated the effectiveness of inositol supplementary for prevention of GDM in women with PCOS (D'Anna et al. 2012), and overweight women (D'Anna et al. 2015). However, all previous studies were carried out in Europe in Caucasian populations. Participants in most previous studies were administered with myo‐inositol (MI), whereas the isomer D‐chiro inositol (DCI) has been less studied.

China has faced a high burden of GDM, with GDM prevalence reported to have increased from 11.4% to 14.5% between 2008 and 2017 in the Chinese population (Gao et al. 2019; Lin et al. 2022). Effective preventive measures are urgently required. Our aim of this study is to investigate the preventative effect of DCI in pregnant women with high risk in the Chinese population.

## 2 Material and Methods

### 2.1 Study Participants and Settings

This double‐blind, randomized clinical trial was carried out in two centers: a tertiary hospital referral center, Women's Hospital of Zhejiang University School of Medicine (average annual birth rate 20,000), and a regional hospital, Quzhou Maternal and Children's Hospital (average annual birth rate 3500), both in Zhejiang Province, China. The recruitment was carried out between January 2020 and December 2022. High‐risk pregnant women with a gestational age of 12–16 weeks and willing to participate were enrolled at the first antenatal visit. A woman was considered high risk if her age was ≥ 35 years old, she had a pre‐pregnancy body mass index (BMI) ≥ 24 kg/m^2^, had a history of GDM, PCOS, or delivering macrosomia infants, or she possessed a family history of type 2 diabetes. The exclusion criteria were as followings: (1) type 1 or type 2 diabetes mellitus diagnosed before pregnancy, (2) multiple gestation, (3) mental or cognitive impairment that prevented completion of the study.

### 2.2 Randomization and Allocation

Participants meeting the inclusion criteria were allocated to either the intervention or the control group in a 1:1 ratio, according to a random number generated through the central randomization by computer at the Women's Hospital,We also registered at the clinical trial (NCT 04801485). Zhejiang University School of Medicine. Interactive web‐based randomization was applied, and enrolling centers received the allocated randomization numbers after confirming eligibility. The allocations were sealed at the enrollment office and remained sealed until the primary outcomes occurred. Both clinical doctors and participants were blinded to the group assignments. Should adverse events arise, or participants request to withdraw from the trial, the investigator would uncover the blinding. Participants were also withdrawn if diagnosed with GDM and needed pharmacological or insulin treatment before the oral glucose tolerance test (OGTT).

The intervention Group received 500 mg of DCI (Openature Amicogen Inc., Jinju, Korea) twice daily, as the Control Group received a placebo with similar appearance and smell but lacking inositol. All participants received either DCI or placebo for 12 weeks, from recruitment to the OGTT, accompanied by consistent advice on diet and exercise. All underwent standard prenatal examinations. Clinicians and participants remained blinded to the allocation.

### 2.3 Outcomes

The primary outcome measure was the occurrence of GDM in both groups. Per the IADPSG guidelines, a diagnosis of GDM is performed with a 75‐g, 2‐h oral glucose tolerance test between 24–28 gestational weeks. The diagnostic thresholds are set at 92 mg/dL (5.1 mmol/L) for time 0, 180 mg/dL (10.0 mmol/L) after 1 h, and 153 mg/dL (8.5 mmol/L) after 2 h (International Association of Diabetes and Pregnancy Study Groups Consensus Panel et al. 2010). Secondary outcomes included perinatal outcomes such as maternal weight gain, neonatal weight, mode of delivery, hypertensive disorders, preterm delivery, shoulder dystocia, and postpartum hemorrhage. Hypertension disorders were further categorized based on the severity of gestational hypertension (the transient hypertension of pregnancy) and preeclampsia/eclampsia (“Gestational Hypertension and Preeclampsia: ACOG Practice Bulletin, Number 222,” 2020).

### 2.4 Sample Size Calculation

Considering the 40% incidence of GDM in high‐risk populations (Khalil et al. 2013; Sun et al. 2023), a 50% reduction in occurrence, as well as a 10% loss of follow‐up rate, we estimated 135:135 participants in the control and intervention groups, achieving an 80% power and a significance level of 0.05.

### 2.5 Statistical Analysis

No participants withdrew between the time of randomization and the administration of the first dose of inositol. Therefore, the baseline characteristics of all participants were compared using either the chi‐squared test or Fisher's exact test for categorical variables, or the independent samples *t*‐test for the quantitative variables. Crude relative ratios (RRs) and 95% confidence intervals (CIs) were calculated for primary and secondary outcomes. Owing to the high rate of loss to follow‐up, we calculated the occurrence of outcomes in the population who completed the OGTT test and in the population who completed delivery, respectively, based on the per‐protocol model. No imputation was applied to any missing data. In addition, we also performed a subgroup analysis to assess the treatment effects across various subgroups. Statistical analyses were conducted using SPSS (version 22.0; Chicago, IL, USA) and MedCalc Software version 15.0 (Ostend, Belgium). Graphs and charts were created using GraphPad Prism 8 (GraphPad, San Diego, CA, USA). Statistical significance was established at *p* < 0.05, and all *p*‐values were two‐tailed.

### 2.6 Ethics Approval

This study involving human participants was approved by the Ethics Committee of Women’s Hospital, Zhejiang University of Medicine (IRB‐20190035‐R). We also registered at the clinical trial (NCT 04801485).

## 3 Results

### 3.1 Study Patients and Follow‐Up

Between 2020 and 2022, a total of 276 participants were enrolled, with 139 randomly assigned to the DCI Group, while 137 to the Control Group. In total, 53 participants (19.2%) were enrolled because of advanced age, 175 (63.4%) were pre‐pregnancy overweight, 29 (10.5%) had a family history of diabetes, 31 (11.2%) had a history of GDM, 25 (9.1%) had a history of delivering macrosomia infants, while 20 (7.2%) were diagnosed with PCOS. The two groups were balanced at baseline in terms of maternal age, parity, education level, and assisted reproduction technology (ART) proportion, as well as pre‐pregnancy BMI, family history of diabetes, and previous gestational history (Table 1). The median gestational weeks for enrollment were comparable (median, interquantile range [IQR] 13, [12–13] vs. 12, [11–13], *p* = 0.207).

**TABLE 1:** Baseline characteristics at enrollment.

	Placebo	DCI	*p*
*N*	137	139	
Demographic characteristic			
Maternal age, median (IQR) (missing data = 4)	31 (27, 34)	30 (28, 34)	0.689
Maternal education (missing data = 4)			
Lower than junior high school	19 (14.2%)	26 (18.8%)	0.620
Senior high school	46 (34.3%)	39 (28.3%)	
Undergraduate or higher	69 (51.5%)	73 (52.9%)	
Assisted reproductive technology	9 (6.6%)	10 (7.2%)	0.838
Multipara (missing data = 6)	82 (62.1%)	80 (58.0%)	0.061
Gestation at randomization, median (IQR), w (misssing data = 4)	13 (12, 13)	12 (11, 13)	0.207
High risk factors			
Maternal age ≥ 35	26 (19.1%)	27 (19.4%)	0.902
Maternal BMI			
BMI at enrollment, mean (SD)	25.4 (3.4)	24.9 (3.5)	0.264
BMI < 24	43 (32.3%)	51 (37.5%)	0.374
BMI ≥ 24	90 (67.7%)	85 (62.5%)	
Family history of diabetes	13 (9.6%)	16 (11.6%)	0.584
Past history of GDM	17 (12.5%)	14 (10.1%)	0.538
History of delivering macrosomia	13 (9.6%)	12 (8.7%)	0.804
Past history of PCOS	7 (5.1%)	13 (9.4%)	0.170

Abbreviations: BMI, body mass index; DCI, D‐chiro inositol; IQR, inter‐quartile range.

**FIGURE 1:**
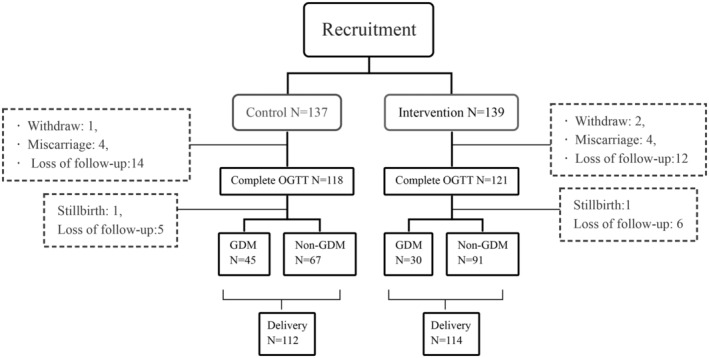
Flow chart.

In the DCI Group, there were four miscarriages, 12 women were transferred to other hospitals, and two women withdrew. In the Control Group, there were four miscarriages, 14 women transferred to other hospitals and one women withdrew. One woman withdrew because of hyperemesis gravidarum, while no participant reported side effects from the drugs. During the second trimester, 121 participants in the DCI Group and 118 in the Control Group underwent an OGTT evaluation. Six participants were lost to follow‐up, and one termination of pregnancy for fetal anomaly (TOPFA) occurred in the DCI Group, while five were loss to follow‐up, and one stillbirth occurred in the Control Group, resulting in 114 women remaining in the DCI Group and 112 in the Control Group finally delivered (Figure 1).

### 3.2 Primary Outcomes

**FIGURE 2:**
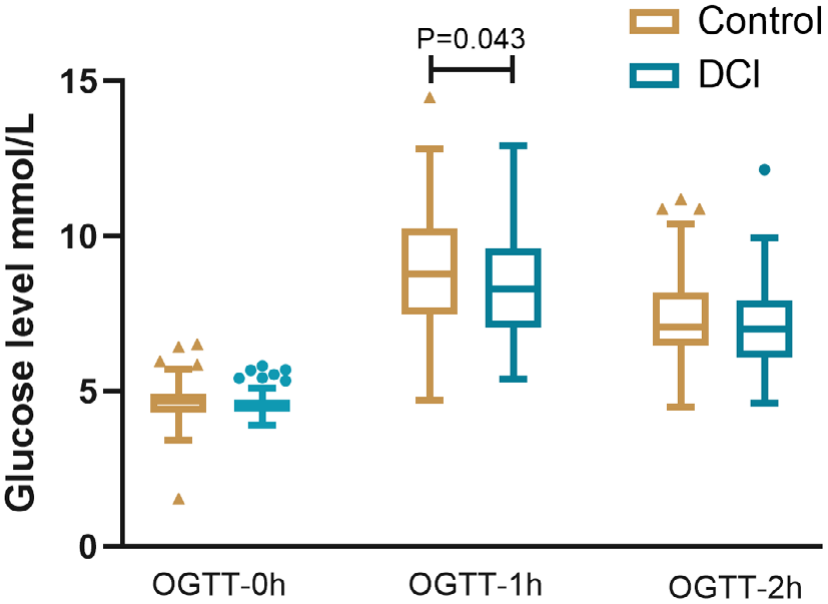
Oral glucose tolerance test at 24–28 gestational weeks between DCI and placebo groups. Fasting glucose, 1‐h and 2‐h glycemia were compared between the DCI and placebo groups. The horizontal lines in the boxes represent the mean, while the lower and upper ends of the boxes represent the 25th and 75th percentiles, respectively. Whiskers represent values within 1.5× the IQR. Data exceeding these values are plotted individually as triangles or circles. DCI, D‐chiro inositol.

**FIGURE 3:**
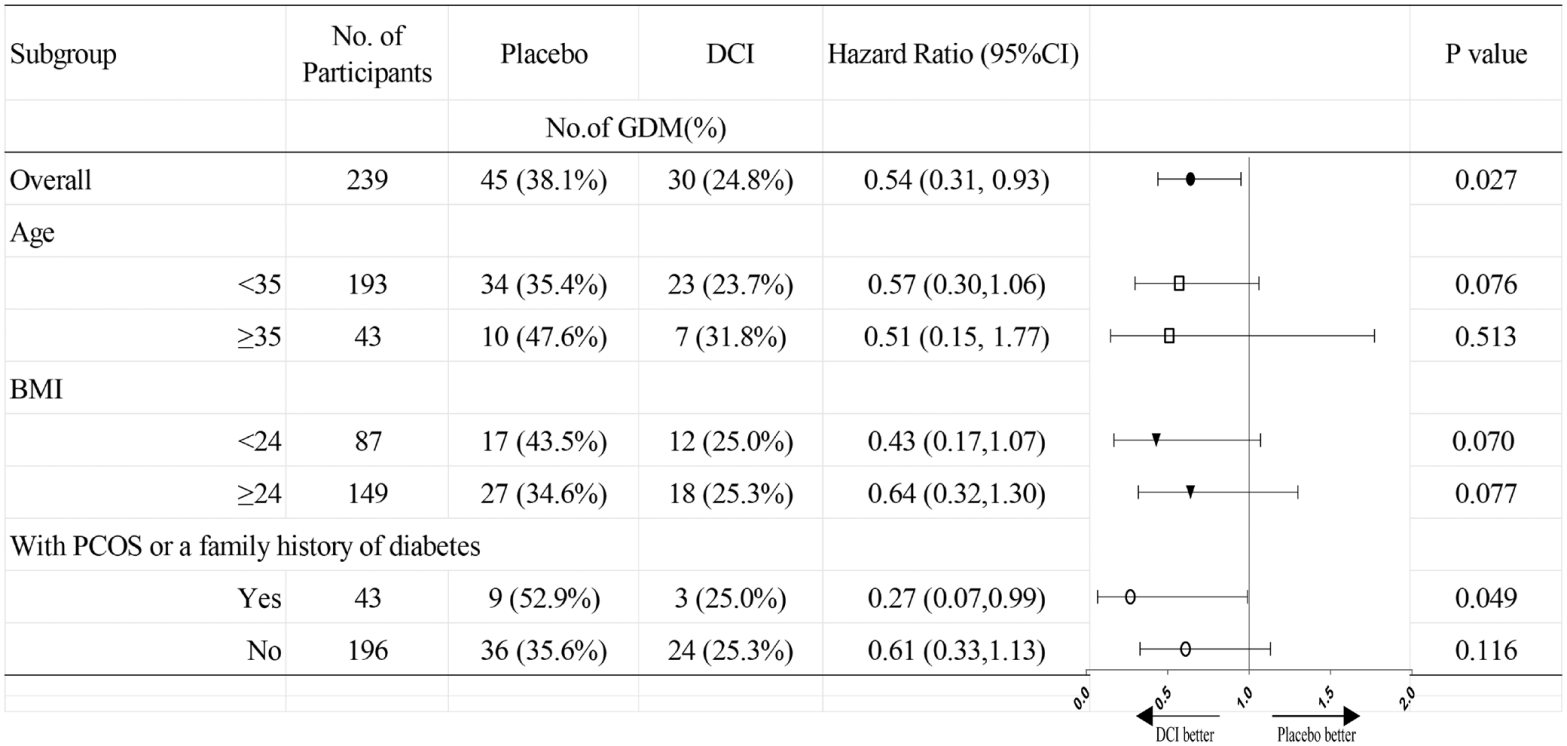
Hazard ratio for the GDM occurrence in prespecified subgroups. CI, confidence interval; DCI, D‐chiro inositol.

GDM occurred in 30 women (24.8%) in the DCI group according to OGTT evaluation, and 45 women (38.1%) in the Control Group (hazard ratio, 0.54, 95% confidence interval [CI] 0.31, 0.93). A significant difference was observed in 1‐h glycemia during OGTT (8.35 ± 1.55 vs. 8.81 ± 1.85, *p* = 0.043, Figure 2), conversely, no difference in fasting glucose level or 2‐h glycemia was found between the two groups. In the subgroup analysis, the reduction in the rate of GDM with DCI was consistent across all major subgroups (Figure 3). However, significant differences were observed only in women either with PCOS or a family history of diabetes (*N* = 43, HR 0.27, 95% CI 0.07, 0.99).

### 3.3 Secondary and Other Efficacy Outcomes

The mean birth weight of newborns in the Control Group was significantly heavier than that of the DCI group (3487.9 ± 437.7 g vs. 3341.6 ± 420.1 g, *p* = 0.011, Table 2). Fourteen (12.5%) neonates weighed > 4000 g in the Control Group compared to six (5.3%) in the DCI Group (*p* = 0.055). There was no difference between the groups in terms of maternal gestational weight gain, hypertension disorders, preterm birth, or delivery mode (Table 2).

**TABLE 2:** Maternal and fetal outcomes between placebo and DCI groups.

	No/Total (%)/Mean (SD)		
	Placebo	DCI	*p*
Primary outcome			
GDM at diagnosis	45/118 (38.1)	30/121 (24.8)	0.026
Secondary outcome			
Maternal weight gain, mean (SD), kg	12.9 (4.6)	12.8 (4.1)	0.826
Hypertension disorders			
None	97/112 (87.4)	104/114 (91.2)	0.579
Gestational hypertension	11/112 (9.9)	7/114 (6.1)	
Preeclampsia	3/112 (2.7)	3/114 (2.6)	
Mode of delivery			
Vaginal delivery	46/112 (41.4)	63/114 (55.3)	0.061
Forceps delivery	2/112 (1.8)	4/114 (3.5)	
Cesarean section	63/112 (56.8)	47/114 (41.2)	
Preterm birth	47/112 (6.3)	7/114 (6.1)	0.973
Shoulder dystocia	2/112 (1.8)	5/114 (4.4)	0.446
Postpartum hemorrhage	8/112 (7.1)	9/114 (7.9)	0.830
Placental abruption	7/112 (6.3)	2/114 (1.8)	0.100
Fetal outcomes			
Neonatal weight, mean (SD), g	3487.9 (437.7)	3341.6 (420.1)	0.011
Macrosomia	14/112 (12.5)	6/114 (5.3)	0.055
Apgar − 1 min ≤ 7	4/112 (3.6)	2/114 (1.8)	0.444

Abbreviations: DCI, D‐chiro inositol.

## 4 Discussion

### 4.1 Main Findings

Our randomized controlled trial demonstrated that intervention with DCI 500 mg twice a day from early pregnancy in women with a high risk for GDM, did reduce the occurrence of GDM (24.8% vs. 38.1%, *p* = 0.027). At OGTT, the mean 1‐h glycemia in the inositol supplementary group was significantly decreased (8.35 ± 1.55 vs. 8.81 ± 1.85, *p* = 0.043). Moreover, compared with the Control Group, the mean birth weight of newborns was lower in the DCI intervention group (3341.6 ± 420.1 g vs. 3487.9 ± 437.7 g, *p* = 0.011).

### 4.2 Interpretations

Consistent with our findings, previous studies demonstrated that inositol supplementation could be a preventive strategy against GDM (Celentano et al. 2020; D'Anna et al. 2015, 2013; Farren et al. 2017; Santamaria et al. 2016; Vitale et al. 2021). All previous researches have focused on pregnant women at high risk for GDM, however, the inclusion criteria have varied significantly. Most of studies targeted pregnant women who are overweight or obese (D'Anna et al. 2015; Santamaria et al. 2016; Vitale et al. 2021), while other studies concentrated on those with a family history of diabetes (D'Anna et al. 2013; Farren et al. 2017). Our study was designed with inclusion criteria that aimed to enroll a broad spectrum of high‐risk groups, (Zhang et al. 2021), giving that the disease's mechanisms differ across various risk factors. It should be noted that the occurrence of GDM in our study was relatively high, with 38.1% in the control group, compared to that in previous studies (D'Anna et al. 2013; Farren et al. 2017; Santamaria et al. 2016), which underscores the effectiveness of our inclusion criteria in turn. Besides, IADPSG 2010 was employed as the diagnosis criteria in our study, which is widely used in Europe and Asia, but distinct from the two‐step criteria used in other regions, potentially affecting GDM incidence rates.

We selected a BMI threshold of ≥ 24 kg/m^2^ for our inclusion criteria, aligning with the definitions of overweight (24 kg/m^2^ ≤ BMI < 28 kg/m^2^) and obesity (BMI ≥ 28 kg/m^2^) in China (Criteria of Weight for Adults. National Health and Family Planning 2013). This threshold in our research may offer better generalizability to the Asian population, contrasting with prior trials primarily involving the European population. Notably, D'Anna et al. (2015) reported a reduction in the GDM rate in the inositol group among women with a BMI ≥ 30 kg/m^2^ (D'Anna et al. 2015), and Santamaria et al. (2016) confirmed similar outcomes in obese women with a BMI ≥ 25 and < 30 kg/m^2^ (Santamaria et al. 2016), nevertheless, our subgroup analysis revealed no significant effect in women with a BMI ≥ 24 kg/m^2^, possibly due to the small sample size post‐subgrouping.

Our findings affirm the benefits of DCI on neonatal weight, corroborated by (D'Anna et al. 2013) in women with a family history of type 2 diabetes. Nonetheless, the difference of birth weight did not influence the rate of macrosomia or the mode of delivery in our study. The relatively high cesarean‐section rate is explained by the substantial number of multiparas with prior cesarean‐section in participants, reflecting the ongoing issue of a high C‐section rate of around 40% in China (Song et al. 2021).

In this study, we investigated the effect of DCI in early pregnancy to prevent GDM, as most of previous studies primarily focused on MI (D'Anna et al. 2015, 2013; Santamaria et al. 2016). It was well‐known that MI and DCI were the predominant isomers of inositol. MI is the most abundant form in human body, while DCI is transformed from MI by an epimerase. Participating with phosphate metabolites, MI was involved in the complex insulin transduction network (Bizzarri et al. 2023), and has been proved to alleviate insulin resistance and sustain metabolic homeostasis. DCI also mediates the action of insulin, besides, its supplementation led to improved luteinizing hormone/follicle stimulation hormone (LH/FSH), and also a better oocyte quality in PCOS women (Cheang et al. 2008; Genazzani et al. 2014).

Previous studies have compared the efficacy of DCI and MI administration. A superior curative effect of DCI over MI was observed in GDM patients, with a decreased HOMA‐IR and fewer adverse maternal and infant outcomes (He et al. 2021). However, research by Celentano et al. (2020) assessed the impact of different stereoisomers of inositol (MI, DCI, MI + DCI or placebo), and observed a benefit in the MI group, but not in the DCI group (Celentano et al. 2020), inconsistent with our results. Notably, the dose of DCI in Celentano's study was merely 500 mg per day, and it remains unclear whether the differing results were dose dependent. On the other hand, DCI administration seems effective only in a specific population. DCI is transformed from MI by an epimerase, a process that is insulin dependent (Genazzani 2020). PCOS patients with insulin resistance exhibit a lower DCI release (Baillargeon et al. 2006), meanwhile, there is an increased urinary excretion of DCI and a higher MI/DCI ratio in patients with diabetes, as well as in those with diabetic first‐degree relatives (Larner 2002). This proves it to a certain extent that compromised expression or synthesis of epimerase enzyme may contribute to the imbalance of MI/DCI and associated insulin insensitivity. Our study population includes a variety of high‐risk groups, which is very suitable for DCI research. Our subgroup analysis further confirmed the DCI's therapeutic effectiveness in women with PCOS or familial diabetes. Several studies have showed solicitude for the MI/DCI ratio, and pointed out that its alteration possibly resulted in diminished or even detrimental effects (Bizzarri et al. 2023). Thus, determining the optimal inositol dose, whether MI or DCI, warrants further investigation.

### 4.3 Strengths and Limitations

Our randomized controlled trial addressed the gap in inositol supplement studies among Asian populations, as prior research predominantly involved Caucasian women. We specifically examined supplementation, which has been infrequently studied in GDM prevention. The longitudinal follow‐up of maternal and fetal outcomes, coupled with the double‐blind design involving both patients and care providers, also made the results robust.

Some limitations should be addressed. First and foremost, the issue of loss to follow‐up cannot be overlooked. Initially, 276 participants were enrolled, however, only 121 in the DCI Group and 118 in the Control Group completed the OGTT. The transfer rate was relatively high, since one of the centers, Quzhou Maternal and Children's Hospital, was a county‐level facility. Moreover, the follow‐up process was also challenged by the coronavirus disease 2019 pandemic, as some women missed regular prenatal check‐ups. Additionally, the slightly high miscarriage rate was also partly influenced by the pandemic. Therefore, an intention‐to‐treat analysis has not been performed in our study owing to the significant loss to follow‐up rate.

Secondly, DCI was the sole intervention in our trial, and we failed to obtain the information of other nutritional supplements from the participants. Nonetheless, common nutritional supplements like vitamin D or folic acid may also affect the incidence of GDM (Godfrey et al. 2021; Yin et al. 2020). Adherence to identical dietary guidance in both groups likely mitigated this limitation.

## 5 Conclusions

Supplementation with 500 mg of DCI twice daily during early pregnancy may reduce the occurrence of GDM in high‐risk populations. A lower mean fetal birth weight was also observed in the DCI‐supplemented group. Given the previous studies on MI in GDM prevention, future investigations into the MI/DCI ratio and supplement dosage are warranted.

## Consent

Consent was obtained from all participants included in the study.

## Conflicts of Interest

The authors declare no conflicts of interest.

## Data Availability

The datasets used and/or analyzed in the current study are available from the corresponding author upon reasonable request.
